# Innate Antimicrobial Defense of Skin and Oral Mucosa

**DOI:** 10.3390/antibiotics9040159

**Published:** 2020-04-03

**Authors:** Philip W. Wertz, Sarah de Szalay

**Affiliations:** 1 University of Iowa, Iowa City, IA 52240, USA; 2 R&D Manager Hygiene Personal Care, Reckitt Benckiser, Parsippany, NJ 07054, USA; Sarah.deSzalay@rb.com

**Keywords:** antimicrobial lipid, antimicrobial peptides, lysozyme, nutrients, permeability barrier, pH

## Abstract

This special issue intends to review and update our understanding of the antimicrobial defense mechanisms of the skin and oral cavity. These two environments are quite different in terms of water, pH, and nutrient availability, but have some common antimicrobial factors. The skin surface supports the growth of a limited range of microorganisms but provides a hostile environment for others. The growth of most microorganisms is prevented or limited by the low pH, scarcity of some nutrients such as phosphorus and the presence of antimicrobial peptides, including defensins and cathelicidins, and antimicrobial lipids, including certain fatty acids and long-chain bases. On the other hand, the oral cavity is a warm, moist, nutrient rich environment which supports the growth of diverse microflora. Saliva coating the oral soft and hard surfaces determines which microorganisms can adhere to these surfaces. Some salivary proteins bind to bacteria and prevent their attachment to surfaces. Other salivary peptides, including defensins, cathelicidins, and histatins are antimicrobial. Antimicrobial salivary proteins include lysozyme, lactoferrin, and lactoperoxidase. There are also antimicrobial fatty acids derived from salivary triglycerides and long-chain bases derived from oral epithelial sphingolipids. The various antimicrobial factors determine the microbiomes of the skin surface and the oral cavity. Alterations of these factors can result in colonization by opportunistic pathogens, and this may lead to infection. Neutrophils and lymphocytes in the connective tissue of skin and mucosa also contribute to innate immunity.

## 1. Permeability Barriers

Epidermal keratinocytes differentiate to produce the stratum corneum, which covers the surface of the skin. This is a physically tough, chemically resistant and relatively impermeable layer consisting of about 16 layers of flattened, hexagonal corneocytes filled with keratin filaments, bounded by a polymerized protein–lipid envelope and surrounded by multilamellar lipid sheets [1,2,3,4,5,6,7]. As cells move off of the basal layer of the epidermis and enter into differentiation as they move toward the surface, they accumulate proteins and lipids [8,9]. These proteins include, among others, keratins, profilaggrin, involucrin, and loricrin [10]. The lipids that accumulate are packaged into small membrane bounded organelles called lamellar granules [11]. The lamellar granules bud off from the Golgi apparatus [12]. In the final stages of differentiation, profilaggrin is dephosphorylated and proteolytically converted into filaggrin [13]. The lamellar granules migrate to the apical end of the cell, and the contents are extruded into the extracellular space [11]. At about this same time, filaggrin causes aggregation of the keratin filaments resulting in flattening of the corneocytes [13]. The loricrin and involucrin are polymerized at the periphery of the cell by action of a transglutaminase to produce a protein envelope, and a layer of long ω-hydroxyceramide molecules becomes covalently attached to the outer surface of the protein layer [14,15,16]. This compound protein–lipid envelope replaces the plasma membrane. Meanwhile, once the keratins have been aggregated, the filaggrin is proteolytically degraded to its component amino acids [10]. This results in a very high osmotic strength within the corneocyte. The lamellar lipids extruded from the lamellar granules are converted into a mixture of ceramides, cholesterol and fatty acids [9]. There is also some cholesterol sulfate present [17]. These lipids spread into broad multilamellar sheets in the intercellular spaces of the stratum corneum and are responsible for the permeability barrier [5,6,7]. The healthy stratum corneum provides a physical barrier that prevents entry of microorganisms, viruses, and microbial products and environmental toxins. What may be the final step of the keratinization process is desquamation, or sloughing off of cells from the skin surface [18]. This is mediated by trypsin-like and chymotrypsin-like serine proteases [19]. Cholesterol sulfate is a serine protease inhibitor. In recessive X-linked ichthyosis the sterol sulfatase that normally degrades cholesterol sulfate as part of the desquamation process is defective resulting in scaly skin [20].

In the oral cavity, the regions of the hard palate and gingiva resemble the epidermis in that they are covered with a keratinizing epithelium that produces a stratum corneum [21]. The events of keratinization in the keratinizing oral regions are generally similar to what takes place in the epidermis; however, the lamellar granules and therefore the intercellular lipids of the stratum corneum are less abundant [22,23]. The protein envelope is less thick and no lipid is attached [24]. These differences result in the keratinized oral regions being about an order of magnitude more permeable than skin [25]. This is, nevertheless, a sufficient barrier to prevent penetration of microorganisms, viruses, and many enzymes and toxins. Unlike the skin, the oral cavity is a wet environment so there is there is no need for a water barrier.

The inside of the lip, the buccal regions, the soft palate and the underside of the tongue are covered by non-keratinizing epithelia [25]. The differentiation process in the non-keratinizing regions is less dramatic in terms of changes in cell shape and lipid and protein content [25]. Roughly the outer third of the epithelium provides a permeability barrier [26]. The formation of this barrier corresponds to extrusion into the intercellular space of the contents of two small membrane bounded organelles called membrane coating granules [27,28]. One of these small organelles has an electron dense core [27]. The other contains lamellar lipid, but it is not organized in the same way as the in the lamellar granules of the keratinizing epithelia [28]. The extrusion of the contents of these membrane coating granules occurs at about two-thirds of the way to the surface. So the outer one-third of the nonkeratinized epithelium provides a permeability barrier. The nonkeratinized regions are as much as twice as permeable as the keratinized oral regions [25]. This increased permeability compared to skin is somewhat offset by faster turnover. The turnover of the epidermis is about 27 days, while the buccal epithelium turns over in about 14 days [21]. The turnover time for epidermal stratum corneum is about 14 days [2,29,30]. The turnover of the buccal barrier would be approximately 4.7 days.

The dorsum of the tongue is covered by a specialized epithelium that is approximated as a mosaic of keratinized and nonkeratinized epithelia [25].

## 2. pH

The pH at the surface of healthy skin ranges from 4.5–5.4 [31]. This relatively low pH limits the range of microorganisms that can grow on the skin surface since most microorganisms grow best in the pH range of 6.5–7.5 [32]. A number of different mechanisms contribute to acidification of the skin surface [33,34]. These include the extrusion of the acidic contents of lamellar granules, liberation of fatty acids from phosphoglycerides upon their extrusion from lamellar granules, production of urocanic acid from histidine after degradation of filaggrin and production of lactic acid via glycolysis in the granular layer [34,35]. The lowering of pH in the upper granular layer of the epidermis is essential for lipid processing by the hydrolytic enzymes delivered to the intercellular space by lamellar granules [11]. These acid hydrolases from the lamellar granules convert a mixture of phosphoglycerides and sphingomyelin into ceramides and free fatty acids necessary for the barrier function of the stratum corneum. In a number of skin conditions where the microbiome is known to be altered, including acne, atopic dermatitis, and psoriasis, the pH is increased [36,37,38].

The pH of saliva in healthy mouths is 7.0 [39]. Accordingly, the pH of the oral cavity permits the growth of a greater variety of microorganisms than the skin surface, and the mouth is one of the most heavily colonized regions of the human body [40].

Two of the most common human illnesses are dental caries and periodontal disease [41]. Caries result from localized acid production by bacteria in biofilm on the surface of teeth. This acid production results from metabolism of carbohydrates [42]. Gingivitis and chronic periodontitis both alter the pH of saliva [39].

Maintenance of an acid pH of the skin has been widely studied and is known to have multiple benefits to the health of the skin. Acidic pH has been shown to accelerate barrier repair in aged skin [43]. The maintenance of an acid pH of the skin has also been linked to the stability and adherence of the skin microbiome. An acid pH has been demonstrated to result in significantly less detachment of skin flora versus alkaline conditions. In some experiments, treatment of skin with an alkaline solution resulted in roughly 10× the counts of residents being removed versus treatment with an acidic solution. It has been further shown that the growth of *Staphylococcus epidermidis* is enhanced in vitro at pH 4.7 as opposed to neutral pH (pH 7). At pH 4.7, this same experiment also showed that the growth of *Staphylococcus aureus* is suppressed [31]. The impact of acidification of the skin has also been evaluated in transmission of illness by hand-to-hand contact. A study done by the University of South Carolina demonstrated that treatment with an acidic cleanser significantly reduced the presence of rhinovirus on the hands of inoculated volunteers at defined time points [44].

## 3. Nutrients

At the surface of the stratum corneum of healthy skin, there is very little phosphorus, iron, or zinc available [45]. This will limit microbial growth. In saliva, lactoferrin sequesters iron, which prevents formation of some microbial biofilms [46].

## 4. Antimicrobial Peptides

Antimicrobial peptides are widely distributed in nature [47]. They are synthesized by keratinocytes in the epidermis and all regions of the oral cavity. These peptides are positively charged and interact with negatively charged phospholipids, lipopolysaccharides (Gram-negative bacteria) and teichoic acids (Gram-positive bacteria) [48]. A hydrophobic portion of the peptide integrates into the membrane lipid, and a pore is formed, thus making the cell leaky.

The most abundant antimicrobial peptides at the skin surface are β-defensins and cathelicidin LL-37. In the oral cavity, β-defensins and cathelicidin LL-37 are also present, but the most abundant antimicrobial peptides in the oral cavity are the histatins. The activity of antimicrobial peptides is sensitive to osmotic strength, and the low osmotic strength of saliva is advantageous in this regard [40].

Antimicrobial peptides can also interact with active immunity by chemotactic and immune-signaling effects [40].

Antimicrobial peptides are also produced by the skin microbiota and are known as bacteriocins. These compounds are also positively charged peptides and are produced by commensal bacteria with broad spectrum and narrow range inhibition spectra. Lantibiotics belong to class I bacteriocins which are a group of ribosomally synthesized, post-translationally modified peptides containing unusual amino acids, such as lanthionine and β-methyllanthionine residues [49]. Lantibiotics are produced by many different types of bacteria (including *S. spp* and *Lactobacillus spp*) and although discovery of new compounds is still emerging, many speculate that this group of compounds holds future potential to treat skin ailments and provide a potential alternative to antibiotics. Some are potent antimicrobials capable of inhibiting biofilm formation. The serine protease Esp2.3 secreted by a subset of *S. epidermidis*, a commensal bacterium, was demonstrated to inhibit biofilm formation and nasal colonization by *S. aureus* [50]. The presence of *S. aureus* has been highly correlated to patients with atopic dermatitis, and further it has been uncovered that these patients also lack the presence of certain isolates of coagulase negative staphylococci. Re-introduction of these isolates from healthy subjects to atopic subjects resulted in a significant decrease in colonization by *S. aureus* and a synergy with LL-37 [51].

## 5. Lysozyme

Lysozyme was discovered by Alexander Fleming in 1922, and is able to kill Gram-positive bacteria by hydrolyzing linkages between N-acetylglucosamine and N-acetylmuramic acid in the peptidoglycan in the cell wall [52,53]. Lysozyme can also kill bacteria independent of its enzymatic activity. This may be due to its positive charge and formation of pores in bacterial membranes [54]. Lysozyme is present in many secretions including saliva [40]. In epidermis lysozyme is found in lamellar granules [54].

## 6. Other Proteins in Saliva

Saliva and other bodily fluids from healthy adults contains thiocyanate (SCN^−^) levels in the range of 1.7 ± 0.8 mM [55]. Hydrogen peroxide is produced in the oral cavity by bacteria of the Streptococcus genus and is in the range of 8–13 µM [56]. Lactoperoxidase uses hydrogen peroxide to oxidize thiocyanate to hypothiocyanate (OSCN^−^) [57]. Hypothiocyanate is antimicrobial, but not against the Streptococcus species producing the hydrogen peroxide [57]. The hydrogen peroxide is also antimicrobial, but not against the *S. spp*.

The flow of saliva from the mouth to the stomach is a major factor protecting the oral mucosa and teeth from adverse microbial activity. The salivary mucin, MUC7, aggregates a wide range of microorganisms, thus preventing them from adhering to mucosal or tooth surfaces [40]. As the saliva is swallowed these organisms will be carried to the acid environment of the stomach.

Likewise, secretory IgA in saliva binds to various microorganisms preventing them from attaching to oral surfaces and allowing them to flow with saliva to the stomach [58].

## 7. Antimicrobial Lipids

That lipids at the skin surface have antimicrobial activity has long been recognized [59]. The short odd carbon fatty acids (C7:0, C9:0, C11:0, C13:0) derived from human sebaceous triglycerides have been identified as antifungals effective in treating ringworm of the scalp [60,61]. Free long-chain bases have been found to be potent and widely active Gram-positive and Gram-negative bacteria [62,63]. Lauric acid (C12:0) and sapienic acid (C16:1Δ6) have been found to have unique antimicrobial activity [63,64]. The same lipids found at the skin surface are also found in the oral cavity [65,66].

## 8. Highlights

In this special issue, it is shown that a delayed skin rash following treatment with gemifloxacin may be mediated by neutrophils [67]). It is shown that human β-defensin 3 (HBD3) induces programmed death-ligand 1 in head and neck squamous cell carcinoma-derived cell lines [68]. Antimicrobial lipids are extensively reviewed [69]. A regional skin microbiome is documented and shown to not alter with use of a lactic acid-containing skin cleansing product, and other skin parameters are measured [70]. It is shown that the distinction between commensal and pathogenic microorganisms on the skin is not always so clear [71]. A novel approach is described for treating infections when the innate immunity of the skin fails [72].
